# Effect of aging on microshearing bond strength of different adhesive systems

**DOI:** 10.1186/s12903-026-08010-5

**Published:** 2026-04-06

**Authors:** Cansu Dağdelen Ahısha, Mine Betül Üçtaşlı

**Affiliations:** Department of Restorative Dentistry, Faculty of Dentistry, Gazi University, Bişkek St. 1.St. Number:8 Emek, Ankara, Turkey

**Keywords:** Adhesive systems, Bond Strenght, Thermal aging

## Abstract

**Background:**

Reliable bonding agents are essential for durable tooth-colored restorations. This study aimed to evaluate the effects of a two-step self-etch adhesive and a universal adhesive (used in self-etch and etch-and-rinse modes) on dentin microshear bond strength and the impact of 1-year simulated thermal aging.

**Methods:**

In this study, 60 caries-free human incisors were used. A two-step self-etch adhesive system (SE) (Clearfil SE Bond; Kuraray Noritake Dental, Japan), a universal adhesive in self-etch mode (U-SE) (Clearfil S3 Bond Universal; Kuraray Noritake Dental, Japan), and the same universal adhesive in etch-and-rinse mode (U-ER) were tested. The specimens were randomly divided into six groups (*n* = 10 per group): (1) SE, initial measurement without aging (T0); (2) U-SE, initial measurement without aging (T0); (3) U-ER, initial measurement without aging (T0); (4) SE, after artificial aging (T1); (5) U-SE, after artificial aging (T1); and (6) U-ER, after artificial aging (T1). Artificial aging was performed by 10,000 thermal cycles between 5 °C and 55 °C, simulating approximately one year of intraoral conditions. The prepared samples were evaluated using the microshear bond strength test. Non-parametric tests were applied for statistical analysis due to the small sample size and non-normal data distribution, with significance set at *p* < 0.05.

**Results:**

The SE without aging group 1 (15.70 MPa) and SE aged group 4 (11.82 MPa) showed the highest bond strength values, while the U-ER without aging group 3 and aged group 6 consistently presented the lowest (6.58 MPa, 6.33 MPa). After 1-year artificial aging, bond strength significantly decreased in the SE (p<0.05) and U-SE (p<0.05) groups, whereas no significant change was observed in the U-ER group (p>0.05). Overall, statistical analysis confirmed significant differences among the adhesive systems and aging conditions (p<0.05).

**Conclusions:**

According to the results of this study, when the micro shear bond strengths of dentin adhesive systems were compared, it was found that the SE group showed higher bond strength values than the U-ER and U-SE groups at the beginning and after 1-year aging simulation.

**Supplementary Information:**

The online version contains supplementary material available at 10.1186/s12903-026-08010-5.

## Background

To meet the increasing aesthetic demands of patients, direct restoration applications with easy-to-apply tooth-colored restorative materials become widespread. Adhesive systems are critical to the success of esthetic restorative materials. Since Buonocore achieved the first successful adhesion to acid-etched enamel surfaces in 1954, the bonding mechanism to enamel and dentin has been extensively investigated and three-step adhesive systems were developed in the early 1990s. To reduce the clinical application time, 2-step etch-and-rinse (ER) and one-step self-etch (SE) adhesive systems were introduced as new generation dental adhesive systems, respectively [1].

Two-step ER adhesive systems consist of the application of a hydrophilic primer and adhesive resin with acid that roughens the surface. SE adhesive systems are known as adhesive systems that do not use a separate acid etching step. SE adhesive systems gently decalcify superficial hydroxyapatite at the enamel and dentin surface and integrate the residual smear layer into the adhesive interface. The depth of decalcification depends on the acidity of the primer: ultra-mild (pH ≥ 2.5) mild (pH ≈ 2), moderately strong (pH 1–2) and strong (pH < 1). The SE adhesive system application is carried out in one or two steps [2].

Adhesive systems called “universal adhesive” or “multi-mode” were developed to combine the “simplification” of the SE technique and the “bond strength” of the ER technique. Universal adhesive systems allow the use of techniques such as ER, SE and selective-etch [3–6].

The structure and natural wetness of dentin make it a difficult substrate for adhesion. In addition, in most clinical situations, dentin remains covered with a smear layer, which prevents the penetration of molecules for adhesion. Therefore, removing the smear layer by acid application (etch-and-rinse system) or modification (self-etch system) is crucial for a strong adhesion [7].

Aging procedures are carried out using mechanical, thermal, or chemical methods, or through combinations of these approaches [8]. Thermal cycling test is a commonly used artificial aging method to simulate the physiological aging of restorative materials. Accelerated water diffusion and temperature fluctuations can weaken the bond strength at the adhesive–material interface [9]. Thermal aging is performed in cycles between 5 °C and 55 °C to simulate thermal changes in the oral environment in vitro [10–12]. 10,000 thermal cycles are considered to be equivalent to approximately 1 year of in vivo thermal cycling [13–15].

Bond strength tests can be categorized based on the direction of the applied force: tensile or shear bond strength tests. Moreover, considering the bonded area, they can be further classified as macro- or micro-tensile and macro- or micro-shear bond strength tests [16]. The micro-shear bond strength test offers several benefits compared to other bond strength methods [16]. It allows multiple samples to be taken from a single tooth and enables testing on very small areas of different dental tissues, such as carious dentin, sclerotic dentin, and enamel [16]. Because the bonded area is minimal, the risk of imperfections like microgaps or air bubbles is reduced [16]. Additionally, the small size of the bonding area facilitates detailed examination using SEM [16].

The aim of this study was to evaluate the effects of a two-step self-etch adhesive system and a universal adhesive system used as self-etch application mode and etch-and-rinse application mode on dentin microshear bond strengths, and the effect of thermal aging process simulating 1-year aging procedures on dentin microshear bond strength.

The first null hypothesis is that there is no difference in dentin bond strength among different adhesive systems. The second null hypothesis is that there is no difference in dentin bond strength among different application modes of the same adhesive system. The third null hypothesis is that the dynamic aging process has no effect on dentin bond strength.

## Methods

The study was approved by the Clinical Research Ethics Committee of Gazi University Faculty of Dentistry, Ankara, Turkiye (Protokol number: GÜDHKAEK.2022.04/2).

Gazi University Scientific Research Projects Unit supported this thesis study. The study was conducted in Gazi University Faculty of Dentistry, Department of Restorative Dentistry Research Laboratory.

### Specimen preparation

In the study, 60 extracted non-caries human incisor teeth were used. The teeth were stored in saline solution no longer than 3 months. The roots of the teeth were separated 1–2 mm apical to the enamel-dentin junction. Then, the teeth were placed in autopolymerized acrylic resin surrounded by a polyvinylchloride (PVC) cylinder, with the buccal surfaces on top. The buccal surfaces of the teeth were grounded wet to expose the dentin using 180-grit silicon carbide abrasive paper with a polishing machine (Mecapol P230, Presi, Turkey) (Fig. 2A). The exposed dentin surfaces of the teeth were polished for 60 s using 320-grit silicon carbide paper under water cooling to create a standard smear layer (Fig. 2B).

Prior to group allocation, a power analysis was performed to determine the required sample size. Based on an effect size of 0.5, α = 0.05, and a desired power (1-β) of 0.80, the total sample size was calculated as 48, requiring at least 8 teeth per group. In the study, 10 teeth were assigned to each group.

**Fig. 1:**
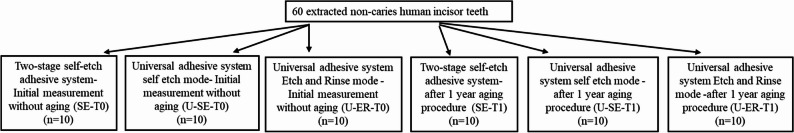
Study Design Overview

In the study, the teeth to be tested were randomly divided into 6 groups (Table 1; Fig. 1). Then the composite resin restorative material applied following the application of the two-step self-etch adhesive system, the universal adhesive system used as self-etch mode and etch-rinse mode was evaluated for the microshear bond strength.

**Table 1:** Grouping of Test Samples

Groups	Procedures Applied to Samples
1 (SE-T0)	Two-stage self-etch adhesive system (Initial measurement without aging)
2 (U-SE-T0)	Universal adhesive system self etch mode (Initial measurement without aging)
3(U-ER-T0)	Universal adhesive system Etch and Rinse mode (Initial measurement without aging)
4 (SE-T1)	Two-stage self-etch adhesive system (after 1 year aging procedure)
5 (U-SE-T1)	Universal adhesive system self etch mode (after 1 year aging procedure)
6 (U-ER-T1)	Universal adhesive system Etch and Rinse mode (after 1 year aging procedure)

### Application of adhesive systems

In the 2-step self-etch adhesive system application, the primer was first applied to the dentin (Clearfil SE Bond, Kuraray Noritake Dental, Japan) with a micro brush, waited for 20 s, and the bond was applied and polymerized with 10 s with LED light curing unit (1400mW/cm2, D-Light Pro, GC, Japan) (Table 2).

**Table 2:** pH Level, Composition and Application Protocol of Adhesive Systems Used

Adhesive	Manufacturer	pH	Composition		Lot Number	Application Method
			Primer	Bond		
Clearfil SE Bond	Kuraray Noritake Dental, Japan	2.0	MDP, HEMA, Hydrophilic aliphatic dimethacrylate, dl-camphorquinone, N, N-diethanol-p-tolidine, Water	MDP, Bis-GMA, HEMA, Hydrophobic aliphatic dimethacrylate, dl-camphorquinone, N, N-diethanol-p-tolidine, Colloidal silica	Lot 000404	1- The primer is applied to the surface and left on the surface for 20 s; 2- Dry with light air; 3- Bond is applied and spread over the surface; 4-Polymerization with light cure for 10 s
Clearfil S^3^ Bond Universal	Kuraray Noritake Dental, Japan	2.7	10-MDP, Bis-GMA, HEMA, hydrophobic dimethacrylate, camphorquinone, ethanol, water, silanated colloidal silica		Lot 000041	SE MOD 1-Application for 20 s; 2-Drying with air water spray for 5 s; 3-Polymerization with light cure for 10 s ER MOD 1–15 s orthophosphoric acid gel application (dentin); 2–15 s washing and light air drying; 3-Application for 20 s; 4-Drying with air water spray for 5 s; 5-Polymerization with light cure for 10 s

In the universal adhesive self-etch mode application (Clearfil S [3] Bond Universal, Kuraray Noritake Dental, Japan), the bonding agent was applied to the dentin surface with a micro brush for 10 s and spread on the surface with gentle air until the bonding agent did not move on the surface (not exceeding 5 s). It was polymerized with 10 s with LED light curing unit (Table 2).

In the universal adhesive etch-and-rinse mode application, orthophosphoric acid gel (35% phosphoric acid, K-etchant gel, Kuraray Noritake Dental, Japan) was applied to the dentin surfaces for 15 s, and the acid gel was removed by applying water spray and gently dried with air spray. The bonding agent (Clearfil S3 Bond Universal, Kuraray Noritake Dental, Japan) was applied to the dentin surface for 10 s with a micro brush and spread on the surface with the application of light air until the bonding agent did not move on the surface and then light cured for 10 s with LED light curing unit (Table 2).

### Composite application to samples

**Fig. 2:**
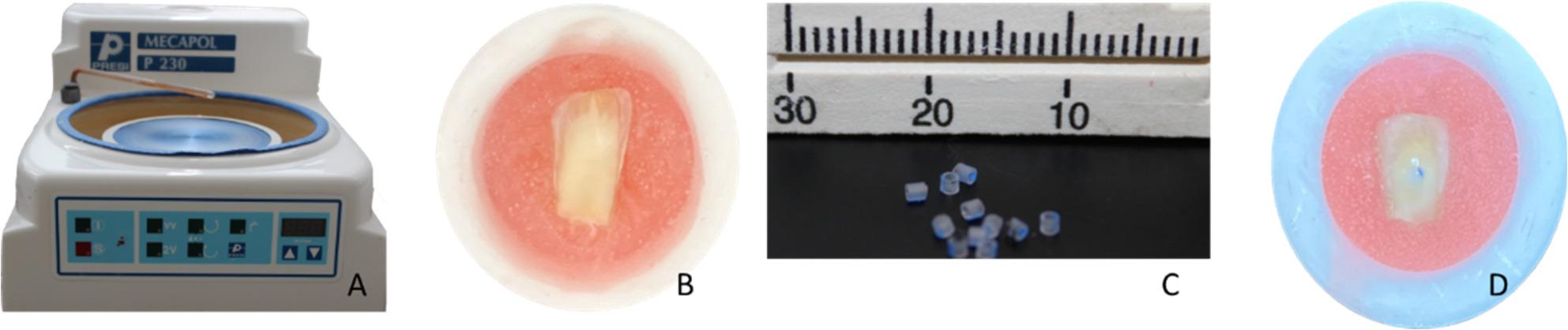
**A**: Sanding and polishing device (Mecapol P230, Presi, Turkey) **B**: Image after sanding the dentin surface with 320-grid sandpaper for 60 s to create a standard smear layer **C**: Transparent tube **D**: Test sample with adhesive and composite resin restorative material

Nanohybrid composite resin restorative material (Clearfil Majesty ES-2 Classic, Kuraray Noritake Dental, Japan) was placed on the dentin surface with the disk-shaped transparent tubes (Nutrition catheter, Bıçakcılar Medical Devices Industry and Trade Inc., Esenyurt, Istanbul, Türkiye) of 0.8 mm inner diameter, 1 mm outer diameter and 1 mm height (Fig. 2C). The inner diameter of the tube defined the bonding area. After applying the adhesive, the transparent tube was positioned and light-cured for 10 s. Subsequently, the composite resin was carefully placed into the transparent tube using a blunt-tip probe and light-cured for 20 s (Fig. 2D). The composition and filler ratio of the composite resin used in this study are presented in Table 3.

**Table 3:** Composition and Filler Ratio of Composite Resin Restorative Material Used

Materiel	Manufacturer	Composition/Particle Size	Filler Ratio (% weight-% volume)	Lot Number
CLEARFIL MAJESTY ES-2	Kuraray Medical Inc., Okayama, Japan	Bis-GMA, Silane barium glass filler, 0.37–1.5 μm (%W)	78%- 40%	C80116

### Application of thermal cycle to samples

The prepared 1st, 2nd and 3rd test groups were stored in water at 37℃ for 24 h without any aging process. The 4th, 5th and 6th test groups were subjected to a thermal aging process of 10,000 times, simulating a 1-year intraoral aging process.

**Fig. 3:**
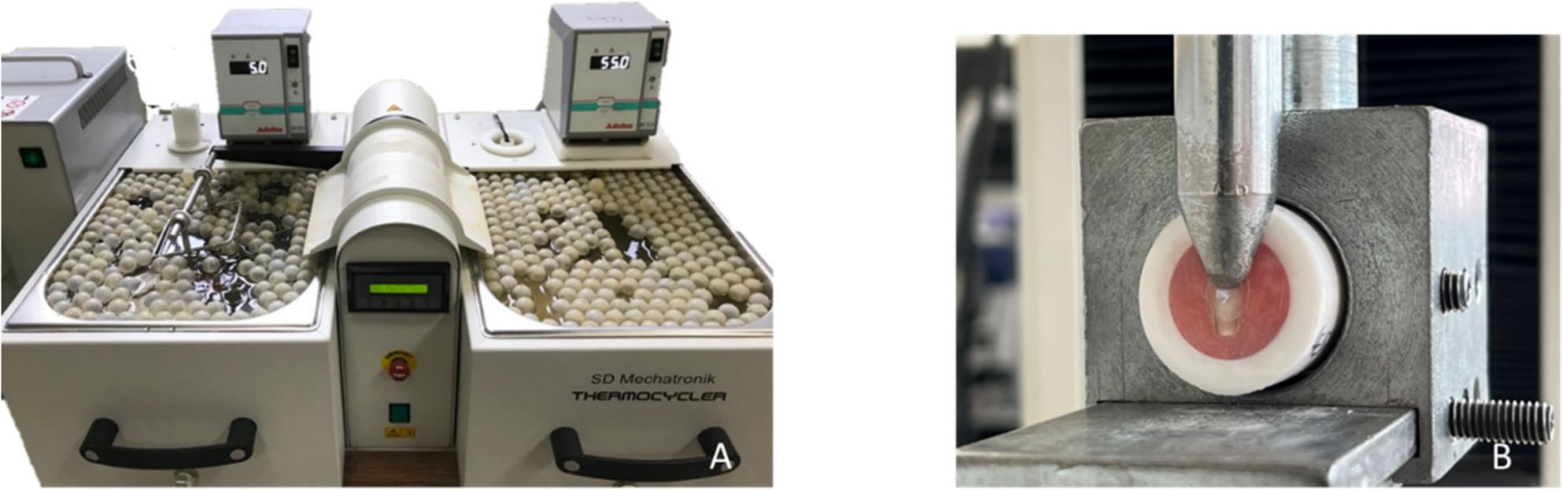
**A**: Thermal cycle device (Thermocycler, SD Mechatronik, Germany) **B**: Fixing the samples to the universal testing machine (Schimadzu IG-IS, Japan) with a special apparatus for microshear bond strength testing.

The termal aging process of the samples was conducted in a standard thermal cycling device (Modental, Esetron Mechatronic, Turkey) (Fig. 3A) at temperatures of 5–55 °C, with a dwell time of 20 s and a transfer time of 10 s.

### Microshear bond strength test

The samples without the aging process, the 1st, 2nd and 3rd group test samples, were stored in water at 37℃ for 24 h. At the end of 24 h, they were fixed to the universal testing machine (Shimadzu IG-IS, Japan) with a special apparatus for microshear bond strength testing (Fig. 3B). The aged 4th, 5th and 6th group test samples were fixed to the universal testing machine with a special apparatus for microshear bond strength testing after being subjected to thermal cycling.

A chisel-shaped blade was positioned perpendicular to the bonding interface between the composite resin and the teeth. The bond strength values, expressed in Newtons (N), were obtained by applying a force at a 1 mm/min rate until failure occurred for each specimen. The obtained N values ​​were divided by the surface area of ​​the samples, converted to Megapascals (MPa) values, and the obtained data were recorded for statistical analysis.

### Statistical analysis

Data analysis was carried out using the SPSS 26 program with a 95% confidence level. Mean, standard deviation (sd), minimum, maximum and median (Min-Max(M)) statistics were given for the measurements. Since the sample size per group was less than 30 and normality was not confirmed (Shapiro-Wilk test, *p* < 0.05), non-parametric tests were used. In the study, Kruskal Wallis test was used to compare measurements by groups, Mann Whitney test was used to compare multiple groups, and Wilcoxon test was used to compare baseline (T0) and 1 year post-aging (T1) measurements for group separation. All statistical comparisons were reported with corresponding p-values, and significance was set at *p* < 0.05.

## Results

According to Kruskal Wallis test results (Table 4), there is a statistically significant difference between the material usage mode groups in terms of initial bond strength (T0) measurements (*p* < 0.05). The T0 measurement is the highest (15.70) in the two-step SE Bond (SE) group and is the lowest (6.58) in the S3 Bond Universal ER mode group (U-ER). According to Mann-Whitney test results (Table 4), there is a statistically significant difference between the SE, S3 Bond Universal in self-etch mode (U-SE) and U-ER groups in terms of T0 measurement (*p* < 0.05).

**Table 4:** Comparison of Initial Bond Strength Measurement by Groups

		T0		SE-USE p	SE-UER p	USE-UER p
		Avg ± SD	Min-Max (M)			
Material-Use Mode p 0,000*	SE	15,70 ± 2,47	12–20 (14)	**0**,006*	**0**,000*	**0**,001*
	U-SE	11,21 ± 1,96	8–14 (11)			
	U-ER	6,58 ± 1,93	5–12 (6)			

*p<0,05 Kruskal Wallis/Mann Whitney

According to Kruskal Wallis test results (Table 5), there is a statistically significant difference between the material usage mode groups in terms of 1-year aging (T1) bond strength measurement (p < 0.05). The T1 bond strength measurement was highest in the SE group (11.82) and lowest in the U-ER group (6.33). According to Mann-Whitney test results (Table 5), there is a statistically significant difference between the SE, U-SE, and U-ER groups regarding T1 bond strength measurement (p < 0.05).

**Table 5:** Comparison of 1-Year Aging Bond Strength Measurement by Groups

		T1		SE-USE p	SE-UER p	USE-UER p
		Avg ± SD	Min-Max (M)			
Material-Use Mode p 0,000*	SE	11,82 ± 1,82	9–14 (11)	**0**,000*	**0**,000*	**0**,049*
	U-SE	9,17 ± 1,64	8–13 (8)			
	U-ER	6,33 ± 1,05	4–8 (6)			

*p<0,05 Kruskal Wallis/Mann Whitney

**Fig. 4:**
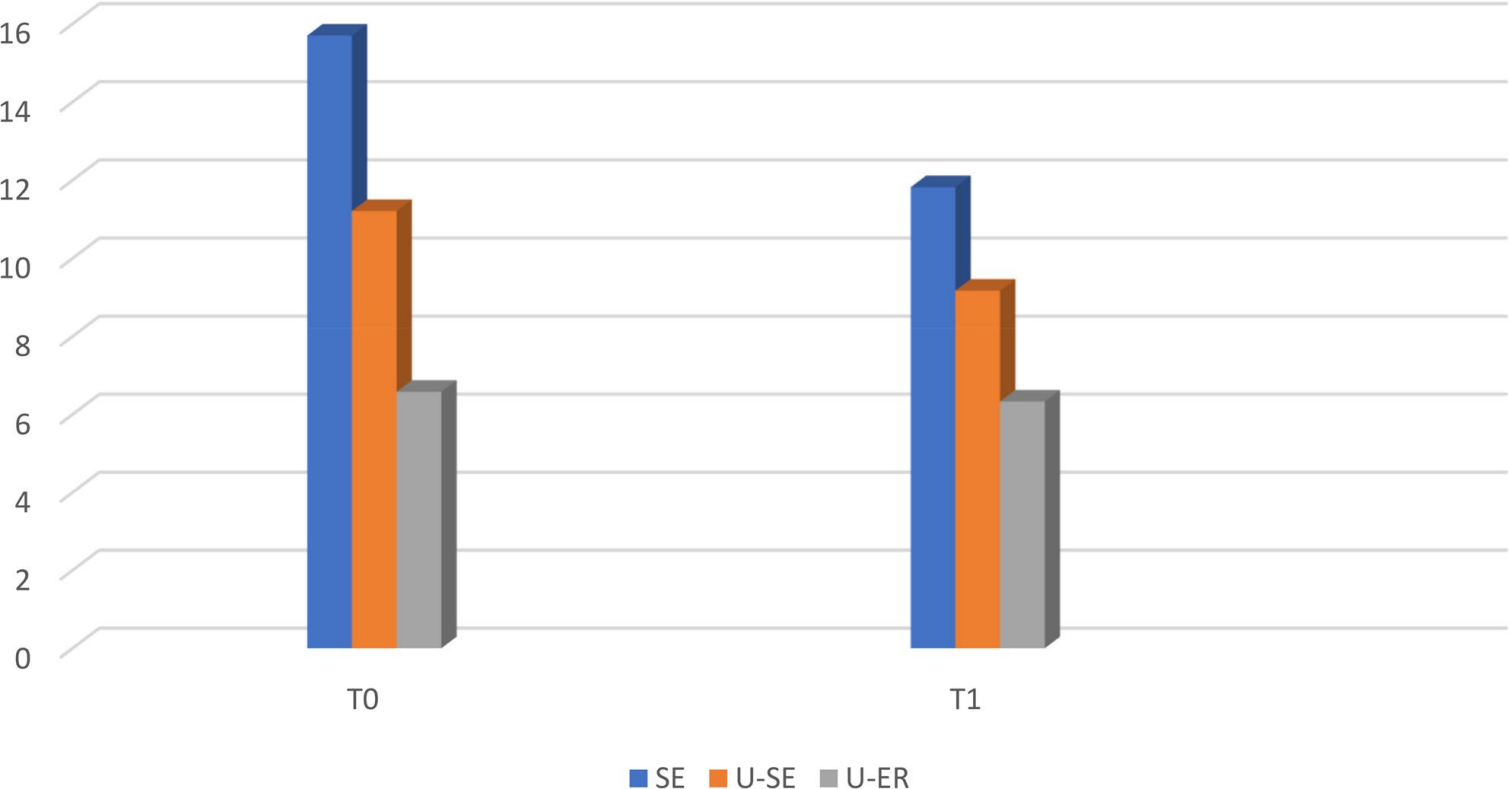
Comparison of T0 and T1 Bond Strength Measurements by Groups

Wilcoxon test results (Table 6; Fig. 4) show a statistically significant difference between bond strength measurements at T0 and T1 in the SE and U-SE groups (*p* < 0.05). T1 bond strength measurement was lower than T0 bond strength measurement in the SE (*n* = 8 samples showed a decrease, Mean = 11.82) and U-SE (*n* = 9 samples showed a decrease, Mean = 9.17) groups. The difference between bond strength measurements of To ad T1 for the U-SE group is not significant (*p* > 0.05).

**Table 6:** Comparison of Bond Strength Measurements in Group Separation between Initial (T0) and 1 Year Aging (T1)

		T0		T1		p
		Avg ± SD	Min-Max (M)	Avg ± ss	Min-Max (M)	
Material-Use Mode	SE	15,70 ± 2,47	12–20 (14)	11,82 ± 1,82	9–14 (11)	**0**,037*
	U-SE	11,21 ± 1,96	8–14 (11)	9,17 ± 1,64	8–13 (8)	**0**,009*
	U-ER	6,58 ± 1,93	5–12 (6)	6,33 ± 1,05	4–8 (6)	**0**,**859**

*p<0,05 Wilcoxon

## Discussion

Based on the results, the first null hypothesis—that there is no difference in dentin bond strength among adhesive systems is rejected, as significant differences were observed (*p* < 0.05), with SE showing the highest and U-ER the lowest bond strength. The second null hypothesis no difference between application modes of the same adhesive is partially rejected, with U-SE and U-ER showing significant differences, while some intra-group changes were not significant. The third null hypothesis—that dynamic aging has no effect is partially rejected, as bond strength decreased significantly from T0 to T1 in SE and U-SE groups (*p* < 0.05), but not in U-ER group.

Dentin is a composite structure consisting of inorganic and organic structures. Structurally, it is a collagen-based tissue consisting of collagen (90%), non-collagenous proteins (10%) and inorganic apatite crystals embedded in the extracellular matrix (ECM), containing 30% (by volume) organic matrix [17].

It is more difficult to achieve ideal bonding in dentin than in enamel tissue. The structure and thickness of the dentin tissue (sclerotic or demineralized), the diameter of the dentinal tubules and their density on the surface, the ratio of peritubular and intertubular dentin, and the presence of the smear layer are the factors that affect adhesion in dentin tissue [18].

Successful adhesion between the restorative material and the dentin surface is a decisive feature for the clinical success of such materials. When examining the adhesion of restorative materials, an important issue to be addressed is the bond strength test methodology. In this study, the microshear bond strength test (µSBS) test was used to evaluate the adhesion of adhesive systems with different applications to the dentin structure.

In this study, the µSBS values ​​obtained from all test groups were lower than those reported in the dentin bond strength literature [19–21]. This finding can be explained by the difference in the teeth used in other studies and their different organic, mineral composition and water content.

Dentin tissue contains different MMPs: collagenase MMP-8 [22], gelatinases MMP-2 and − 9 [23−27], stromelysin MMP-3 [28] and enamelin MMP-20 [29]. These MMPs play an active role during tooth development [30]. Dentin proteases remain structurally stable as long as the dentin tissue is mineralized^31^. MMPs in dentin tissue can also be activated by the acidic properties of adhesive systems.

Mazzoni et al. [23] reported that etch-and-rinse (ER) adhesive systems, and Nishitani et al. [31] reported that self-etch (SE) adhesive systems, can reactivate gelatinases (MMP-2 and MMP-9) and collagenases in demineralized dentin. The study by Lehmann et al. [32] showed that the expression of MMP-2 and pro-MMP-9 in odontoblasts increased after using SE adhesive systems on dentin tissue. Pashley et al. [33] believed that the use of 35–37% phosphoric acid (pH 0.7–1) to acid etch dentin in the ER approach initially denatures MMPs and inactivates pro-MMPs trapped in mineralized dentin due to the low pH. According to the data obtained from this study, in line with the literature, no statistically significant decrease in microtensile bond strength was observed in the U-ER group after aging by thermal cycling.

In this study, both adhesive systems used contain functional monomers such as HEMA, bis-GMA and 10-MDP in their structure. Functional monomers are considered important components of adhesive systems because they regulate the interaction of the adhesive interface with dental tissue. Typically, adhesives contain functional monomers in their composition to increase bond strength, improve diffusion and penetration of other monomers and even provide antimicrobial effect [34].

Jäggi et al. [35], in their study examining the shear bond strength of universal adhesives to enamel and dentin, observed the lowest shear bond strength values in HEMA-free systems and reported no significant decrease in bond strength following thermocycling and aging. Brkanović et al. [36], in their study comparing the shear bond strength of different universal adhesive systems to dentin, found that the HEMA-free universal adhesive, G2-Bond Universal, exhibited the most stable shear bond strength values when applied in the self-etch mode. Takahashi et al. [37], examined the water absorption and tensile bond strength properties of one-step self etch adhesive systems with and without HEMA, and found that water absorption increased, bond strength decreased over time in adhesive systems containing HEMA. Similarly, both adhesive systems evaluated in this study contain HEMA, and this explains the decrease in microshear bond strength values ​​observed in all tested groups in the long term.

The popularity of self etch adhesive systems has increased due to their user-friendly features, resulting in fewer clinical steps and adequate bonding [38]. Such adhesives promote simultaneous demineralization and resin infiltration, lowering the risk of adhesive penetration failure. Self etch adhesive systems reduce the potential problems observed with the ER technique, providing better sealing of the dentinal tubules and creating a more homogeneous hybrid layer, which results in less postoperative sensitivity [39].

However, the life of self etch adhesive systems depends on the level of acidic monomers, as these affect bond strength and stability. Therefore, a mild self etch adhesive is currently recommended for adhesion to dentin [40]. Furthermore, the degradation of collagen fibrils can be reduced by the absence of phosphoric acid application, which directly affects the stability of the bond over time [40]. Raji et al. [41], in their study evaluating the microshear bond strength of mild and ultra-mild universal adhesives to deep dentin, reported that the type of adhesive (mild/ultra-mild) influenced the bond strength to deep dentin, whereas the application mode did not have a significant effect on the microshear bond strength to deep dentin.

Simplifying the clinical application stages of adhesive procedures is targeted as a commercial strategy. For this purpose, adhesive systems called “universal/multimode” have been introduced. Most universal adhesive agents are applied as two-step ER and one-step self etch mode [42–44].

All adhesive systems have their strengths and weaknesses. In general, ER adhesive systems are hydrophobic and therefore durable adhesives, but must be used in conjunction with phosphoric acid etching [7]. This application process is not always the most suitable approach and is considered aggressive to dentin [7]. Self etch adhesive systems avoid phosphoric acid etching of dentin and can be used in conjunction with self etch or selective-etch approaches, but are more hydrophilic and therefore prone to degradation [45]. Finally, universal adhesive agents can be used with or without phosphoric acid etching and have greater hydrophilicity than multi-step adhesives [46]. The bond strength of universal adhesives in self etch and ER modes is debatable [19, 47, 48].

Takamizawa et al. [47], evaluated the bond strength of self etch adhesives to dentin in different modes, reported a decrease in the bond strength of Clearfil SE Bond when applied in etch and rinse mode. Valizade et al. measured the dentine µSBS of Single Bond, Scotchbond Universal and Clearfil SE Bond in ER and self etch modes and reported that the µSBS values ​​were maximum in self etch mode but the difference was not statistically significant. Bakry et al. [49], in their study evaluated the effects of different application modes of two universal adhesive systems on shear bond strength and reported that Tetric N-Bond exhibited the highest shear bond strength when applied in the self etch mode. Hosseini et al. [50] investigated the microshear bond strength of universal adhesives with varying pH levels to superficial dentin in both self etch and etch and rinse application modes. The results of their study demonstrated that All-Bond Universal exhibited the highest microshear bond strength in the self etch mode, whereas the lowest bond strength was observed in the etch and rinse mode. The researchers recommend using the self etch mode because of less demineralization of the dentine surface [51]. According to the results of this study, when the micro shear bond strengths of dentin adhesive systems were compared, it was determined that the SE adhesive system showed higher bond strength values ​​than the U-SE after the initial and 1-year aging simulation. This result can be explained by the fact that the evaluated SE adhesive system has higher acidity than the universal adhesive system. It is thought that the more acidic system among the two adhesive systems, light and ultra-light, causes deeper demineralization on the surface and provides better micromechanical interlocking to the dentin.

## Conclusions

Within the limits of this in vitro study,

- 1-  Two-step SE adhesive groups showed the highest μSBS values among the groups where aging process was not applied or applied.
- 2-   The result of the study showed that the bond strength to dentin was lower in the acid etching protocol.
- 3-   In the two-step SE adhesive group and the group where the universal adhesive was applied in SE mode, μSBS values decreased significantly after aging. No decrease was observed in the μSBS values in the group where the universal adhesive was applied in ER mode.

All these findings should be considered in material selection in clinical applications. In order to investigate the decrease observed in SE and U-SE groups after the aging protocol in more detail, further studies on the subject, both in vitro and in vivo, are needed.

## Supplementary Information

Supplementary Material 1.

Supplementary Material 2.

Supplementary Material 3.

Supplementary Material 4.

Supplementary Material 5.

Supplementary Material 6.

Supplementary Material 7.

## Data Availability

The datasets used and analysed during this study are available from the correspondingauthor upon reasonable request.

## Abbreviations

T0: In the initial bond strength measurement
T1: 1 Year aging applied measurement
SE: The two-stage self-etch SE bond
U-SE: The S3 universal bond used in self-etch mode
U-ER: The S3 universal bond used in etch and rinse mode
ER: Etch and rinse

## References

[1] Tran XV, Tran KQ. Microleakage and characteristics of resin-tooth tissues interface of a selfetch and an etch-and-rinse adhesive systems. Restor Dent Endod. 2021;46:30–43. 10.5395/rde.2021.46.e30 PMC8170381 34123766

[2] Perdigão J, Araujo E, Ramos RQ, Gomes G, Pizzolotto L. Adhesive dentistry: Current concepts and clinical considerations. J Esthet Restor Dent. 2021;33:51–68. 33264490 10.1111/jerd.12692

[3] Muñoz MA, Luque I, Hass V, Reis A, Loguercio AD, Bombarda NHC. Immediate bonding properties of universal adhesives to dentine. J Dent. 2013;41:404–11. 23499568 10.1016/j.jdent.2013.03.001

[4] McLean D, Meyers E, Guillory V, Vandewalle K. Enamel bond strength of new universal adhesive bonding agents. Oper Dent. 2015;40:410–7. 25575201 10.2341/13-287-L

[5] Wagner A, Wendler M, Petschelt A, Belli R, Lohbauer U. Bonding performance of universal adhesives in different etching modes. J Dent. 2014;42:800–7. 24814138 10.1016/j.jdent.2014.04.012

[6] Loguercio AD, Muñoz MA, Luque-Martinez I, Hass V, Reis A, Perdigão J. Does active application of universal adhesives to enamel in self-etch mode improve their performance? J Dent. 2015;43:1060–70. 25908573 10.1016/j.jdent.2015.04.005

[7] Pashley DH, Tay FR, Breschi L, Tjäderhane L, Carvalho RM, Carrilho M, Carrilho M, Tezvergil-Mutluay A. State of the art etch-and-rinse adhesives. Dent Mater. 2011;27:1–16. 21112620 10.1016/j.dental.2010.10.016 PMC3857593

[8] Loomans BAC, Mesko ME, Moraes RR, Ruben J, Bronkhorst EM, Pereira-Cenci T, Huysmans MCDNJM. Effect of different surface treatment techniques on the repair strength of indirect composites. J Dent. 2017;59:18–25. 28174053 10.1016/j.jdent.2017.01.010

[9] Tanaka T, Kamada I, Matsumura H, Atsuta M. A comparison of water temperatures for thermocycling of metal-bonded resin specimens. J Prosthet Dent. 1995;74:345–9. 8531150 10.1016/s0022-3913(05)80372-5

[10] Kitasako Y, Burrow M, Nikaido T, Tagami J. The influence of storage solution on dentin bond durability of resin cement. Dent Mater. 2000;16:1–6. 11203517 10.1016/s0109-5641(99)00061-5

[11] Morresi AL, D’Amario M, Capogreco M, et al. Thermal cycling for restorative materials: does a standardized protocol exist in laboratory testing? A literature review. J Mech Behav Biomed Mater. 2014;29:295–308. 24135128 10.1016/j.jmbbm.2013.09.013

[12] Gale MS, Darvell BW. Thermal cycling procedures for laboratory testing of dental restorations. J Dent. 1999;27:89–99. 10071465 10.1016/s0300-5712(98)00037-2

[13] De Munck J, Vargas M, Van Landuyt K, Hikita K, Lambrechts P, Van Meerbeek B. Bonding of an Auto-Adhesive Luting Material to Enamel and Dentin. Dent Mater. 2004;20:963–71. 15501325 10.1016/j.dental.2004.03.002

[14] De Munck J, Van Landuyt K, Coutinho E, Poitevin A, Peumans M, Lambrechts P, Van Meerbeek B. Micro-tensile bond strength of adhesives bonded to Class-I cavity-bottom dentin after thermo-cycling. Dent Mater. 2005;21:999–1007. 16181669 10.1016/j.dental.2004.11.005

[15] Papacchini F, Toledano M, Monticelli F, Osorio R, Radovic I, Polimeni A, Ferrari M. Hydrolytic stability of composite repair bond. Eur J Oral Sci. 2007;115:417–24. 17850431 10.1111/j.1600-0722.2007.00475.x

[16] Tartıcı T, Gür G. Effect of different cavity disinfectants on the microshear bond strength of universal adhesive applied in different etching modes. Uzmanlık Tezi. Ankara Üniversitesi Diş Hekimliği Fakültesi; 2020.

[17] Butler WT, Brunn JC, Qin C. Dentin extracellular matrix (ECM) proteins: comparison to bone ECM and contribution to dynamics of dentinogenesis. Connect Tissue Res. 2003;44:171–8. 12952193

[18] Mjör IA. Dentin permeability: the basis for understanding pulp reactions and adhesive technology. Braz Dent J. 2009;20:3–16. 19466224 10.1590/s0103-64402009000100001

[19] Takamizawa T, Barkmeier WW, Tsujimoto A, Berry TP, Watanabe H, Erickson RL, et al. Influence of different etching modes on bond strength and fatigue strength to dentin using universal adhesive systems. Dent Mater. 2016;32:9–21. 10.1016/j.dental.2015.11.005 26719131

[20] Daneshkazemi P, Ghasemi A, Daneshkazemi A, Shafiee F. Evaluation of micro shear bonding strength of two universal dentin bondings to superficial dentin by self etch and etch–and–rinse strategies. J Clin Exp Dent. 2018;10:837–43. 10.4317/jced.54740 PMC6203927 30386514

[21] Tokuyama Universal Bond Presentation–Tinman Dental. (2017, May–June). Available from: https://www.tinmandental.com/estore/images/TOKUYAMA–Powerpoint–rs.pdf

[22] Sulkala M, Tervahartiala T, Sorsa T, Larmas M, Salo T, Tjaderhane L. Matrix metalloproteinase-8 (MMP-8) is the major collagenase in human dentin. Arch Oral Biol. 2007;52:121–7. 17045563 10.1016/j.archoralbio.2006.08.009

[23] Mazzoni A, Pashley DH, Nishitani Y, Breschi L, Mannello F, Tjaderhane L, et al. Reactivation of inactivated endogenous proteolytic activities in phosphoric acid-etched dentine by etch-and-rinse adhesives. Biomater. 2006;27:4470–6. 10.1016/j.biomaterials.2006.01.040 16687171

[24] Mazzoni A, Pashley DH, Tay FR, Gobbi P, Orsini G, Ruggeri A Jr, et al. Immunohistochemical identification of MMP-2 and MMP-9 in human dentin: correlative FEI-SEM/TEM analysis. J Biomed Mater Res A. 2009;88:697–703. 18335530 10.1002/jbm.a.31920

[25] Martin-De Las Heras S, Valenzuela A, Overall CM. The matrix metalloproteinase gelatinase A in human dentine. Arch Oral Biol. 2000;45:757–65. 10869489 10.1016/s0003-9969(00)00052-2

[26] Mazzoni A, Mannello F, Tay FR, Tonti GA, Papa S, Mazzotti G, et al. Zymographic analysis and characterization of MMP-2 and – 9 isoforms in human sound dentin. J Dent Res. 2007;86:436–40. 17452564 10.1177/154405910708600509

[27] Mazzoni A, Papa V, Nato F, Carrilho M, Tjäderhane L, Ruggeri A Jr, et al. Immunohistochemical and biochemical assay of MMP-3 in human dentine. J Dent. 2011a;39:231–7. 21215789 10.1016/j.jdent.2011.01.001 PMC3815524

[28] Mazzoni A, Carrilho M, Papa V, Tjäderhane L, Gobbi P, Di Lenarda R, et al. MMP-2 assay within the hybrid layer created by a two-step etch-and-rinse adhesive: biochemical and immunohistochemical analysis. J Dent. 2011b;39:470–7. 21554921 10.1016/j.jdent.2011.04.004

[29] Sulkala M, Larmas M, Sorsa T, Salo T, Tjäderhane L. The localization of matrix metalloproteinase-20 (MMP-20, enamelysin) in mature human teeth. J Dent Res. 2002;81:603–7. 12202640 10.1177/154405910208100905

[30] Tjaderhane L, Palosaari H, Wahlgren J, Larmas M, Sorsa T, Salo T. Human odontoblast culture method: the expression of collagen and matrix metalloproteinases (MMPs). Adv Dent Res. 2001;15:55–8. 12640741 10.1177/08959374010150011401

[31] Nishitani Y, Yoshiyama M, Wadgaonkar B, Breschi L, Mannello F, Mazzoni A, et al. Activation of gelatinolytic/collagenolytic activity in dentin by selfetching adhesives. Eur J Oral Sci. 2006;114:160–6. 16630309 10.1111/j.1600-0722.2006.00342.x

[32] Lehmann N, Debret R, Romeas A, Magloire H, Degrange M, Bleicher F, et al. Self-etching increases matrix metalloproteinase expression in the dentin-pulp complex. J Dent Res. 2009;88:77–82. 19131322 10.1177/0022034508327925

[33] Pashley DH, Tay FR, Yiu C, Hashimoto M, Breschi L, Carvalho RM, Ito S. Collagen degradation by host-derived enzymes during aging. J Dent Res. 2004;83:216–21. 14981122 10.1177/154405910408300306

[34] Van Landuyt KL, Snauwaert J, De Munck J, Peumans M, Yoshida Y, Poitevin A, Countinho E, Suzuki K, Lambrechts P, Van Meerbeek B. Systematic review of the chemical composition of contemporary dental adhesives. Biomater. 2007;28:3757–85. 10.1016/j.biomaterials.2007.04.044 17543382

[35] Jäggi M, Karlin S, Zitzmann NU, Rohr N. Shear bond strength of universal adhesives to human enamel and dentin. J Esthet Restor Dent. 2024;36:804–12. 38308570 10.1111/jerd.13204

[36] Brkanović S, Klarić Sever E, Vukelja J, Ivica A, Miletić I, Jukić Krmek S. Comparison of different universal adhesive systems on dentin bond strength. Materials. 2023;16:1530. 36837160 10.3390/ma16041530 PMC9963205

[37] Takahashi M, Nakajima M, Hosaka K, Ikeda M, Foxton RM, Tagami J. Long-term evaluation of water sorption and ultimate tensile strength of HEMAcontaining/-free one-step self-etch adhesives. J Dent. 2011;39:506–12. 21575671 10.1016/j.jdent.2011.04.008

[38] Miyazaki M, Tsujimoto A, Tsubota K, Takamizawa T, Kurokawa H, Platt JA. Important compositional characteristics in the clinical use of adhesive systems. J Oral Sci. 2014;56:1–9. 24739701 10.2334/josnusd.56.1

[39] Ahn J, Jung KH, Son SA, Hur B, Kwon YH, Park JK. Effect of additional etching and ethanol-wet bonding on the dentin bond strength of one-step self-etch adhesives. Restor Dent Endod. 2015;40:68–74. 25671215 10.5395/rde.2015.40.1.68 PMC4320279

[40] Rosa WL, Piva E, Silva AF. Bond strength of universal adhesives: A systematic review and meta-analysis. J Dent. 2015;43:765–76. 25882585 10.1016/j.jdent.2015.04.003

[41] Raji Z, Hosseini M, Kazemian M. Micro-shear bond strength of composite to deep dentin by using mild and ultra-mild universal adhesives. Dent Res J. 2022;19:44–50. PMC9338388 35915717

[42] Isolan CP, Valente LL, Munchow EA, Basso GR, Pimentel AH, Schwantz JK, da Silva AV, Moraes RR. Bond strength of a universal bonding agent and other contemporary dental adhesives applied on enamel, dentin, composite and porcelain. Appl Adhes Sci. 2014;2:25–35.

[43] Hanabusa M, Mine A, Kuboki T, Momoi Y, Van Ende A Meerbeek Dentin bonds of universal adhesives B, De Munck J. Bonding effectiveness of a new multi-mode adhesive to enamel and dentine. J Dent. 2012; 40: 475–484. 10.1016/j.jdent.2012.02.012 22381614

[44] Perdigão J, Sezinando A, Monteiro PC. Effect of substrate age and adhesive composition on dentin bonding. Oper Dent. 2013;38:267–74. 23210916 10.2341/12-307-L

[45] Ozer F, Blatz MB. Self-etch and etch-and-rinse adhesive systems in clinical dentistry. Compend Contin Educ Dent. 2013;34:12–4. 23550327

[46] Perdigão J, Araujo E, Ramos RQ, Gomes G, Pizzolotto L. Adhesive dentistry: Current concepts and clinical considerations. J Esthet Restor Dent. 2020;33:51–68. 33264490 10.1111/jerd.12692

[47] Singh K, Naik R, Hegde S, Damda A. Shear bond strength of superficial, intermediate and deep dentin in vitro with recent generation self–etching primers and single nano composite resin. J Int Oral Health. 2015;7:28–32. 26225101 PMC4516063

[48] Frankenberger R, Perdigão J, Rosa BT, Lopes M. No–bottle vs. multi–bottle dentin adhesives – A microtensile bond strength and morphological study. Dent Mater. 2001;17:373–80. 11445203 10.1016/s0109-5641(00)00084-1

[49] Bakry AS, Abbassy MA. Application modes affect two universal adhesive systems’ nanoleakage expression and shear bond strength. BioMed Res Int. 2021;7375779:1–8. 10.1155/2021/7375779 PMC8497110 34631886

[50] Hosseini M, Raji Z, Kazemian M. Microshear bond strength of composite to superficial dentin by use of universal adhesives with different pH values in self-etch and etch & rinse modes. Dent Res J. 2023;20:5–11. PMC9937934 36820140

[51] Valizadeh S, Moradi A, Mirazei M, Amiri H, Kharazifard MJ. Microshear bond strength of different adhesive systems to dentin. Front Dent. 2019;16:265–71. 32342055 10.18502/fid.v16i4.2085 PMC7181353

