# Supplementary figures and images for: Effect of aging on microshearing bond strength of different adhesive systems
Source: BMC Oral Health. 2026 Apr 6;26:957. doi: 10.1186/s12903-026-08010-5 (PMC13234965; doi:10.1186/s12903-026-08010-5)

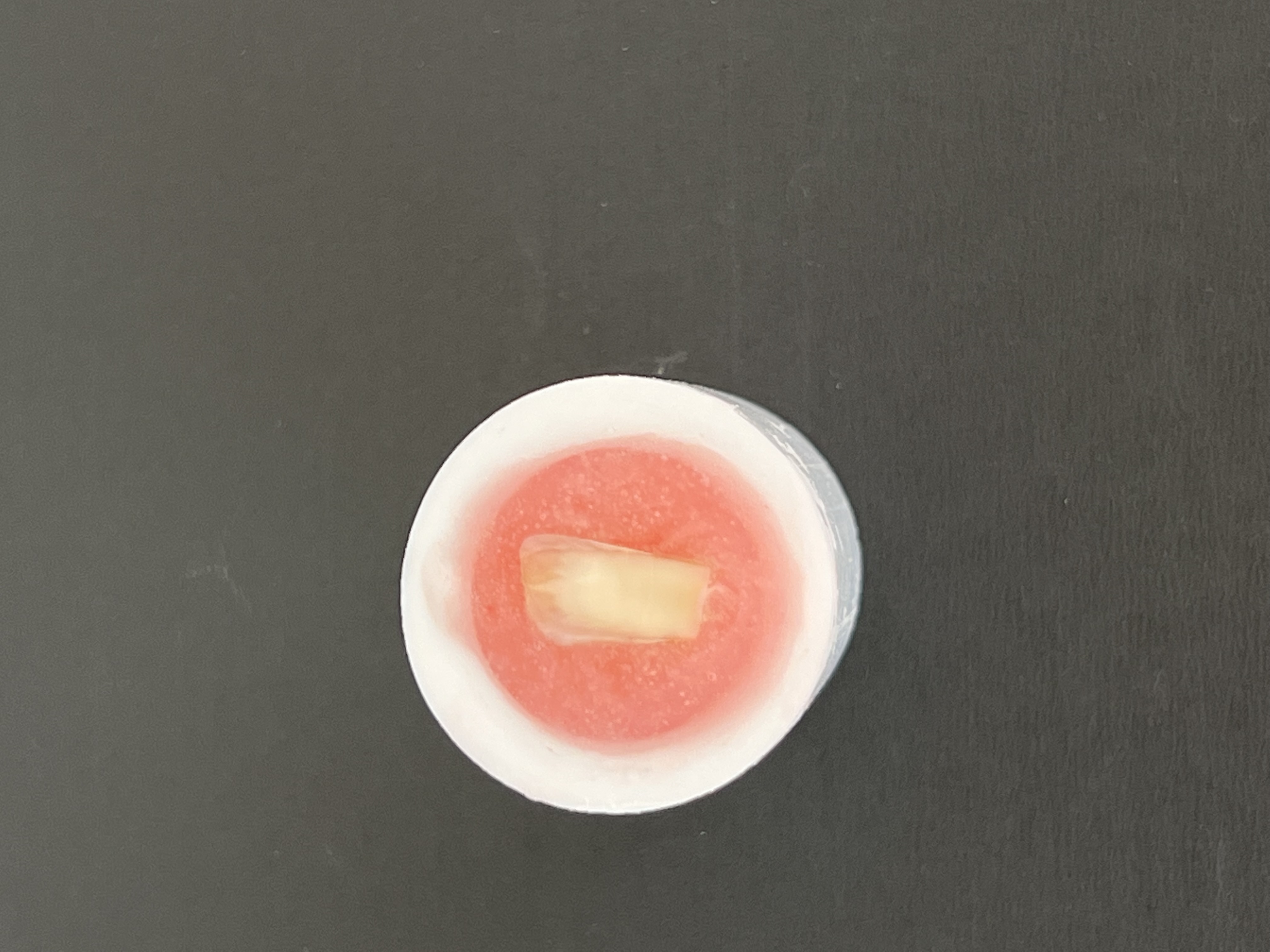

Supplement: Supplementary file 1 — Supplementary Material 1. [file 12903_2026_8010_MOESM1_ESM.jpeg]

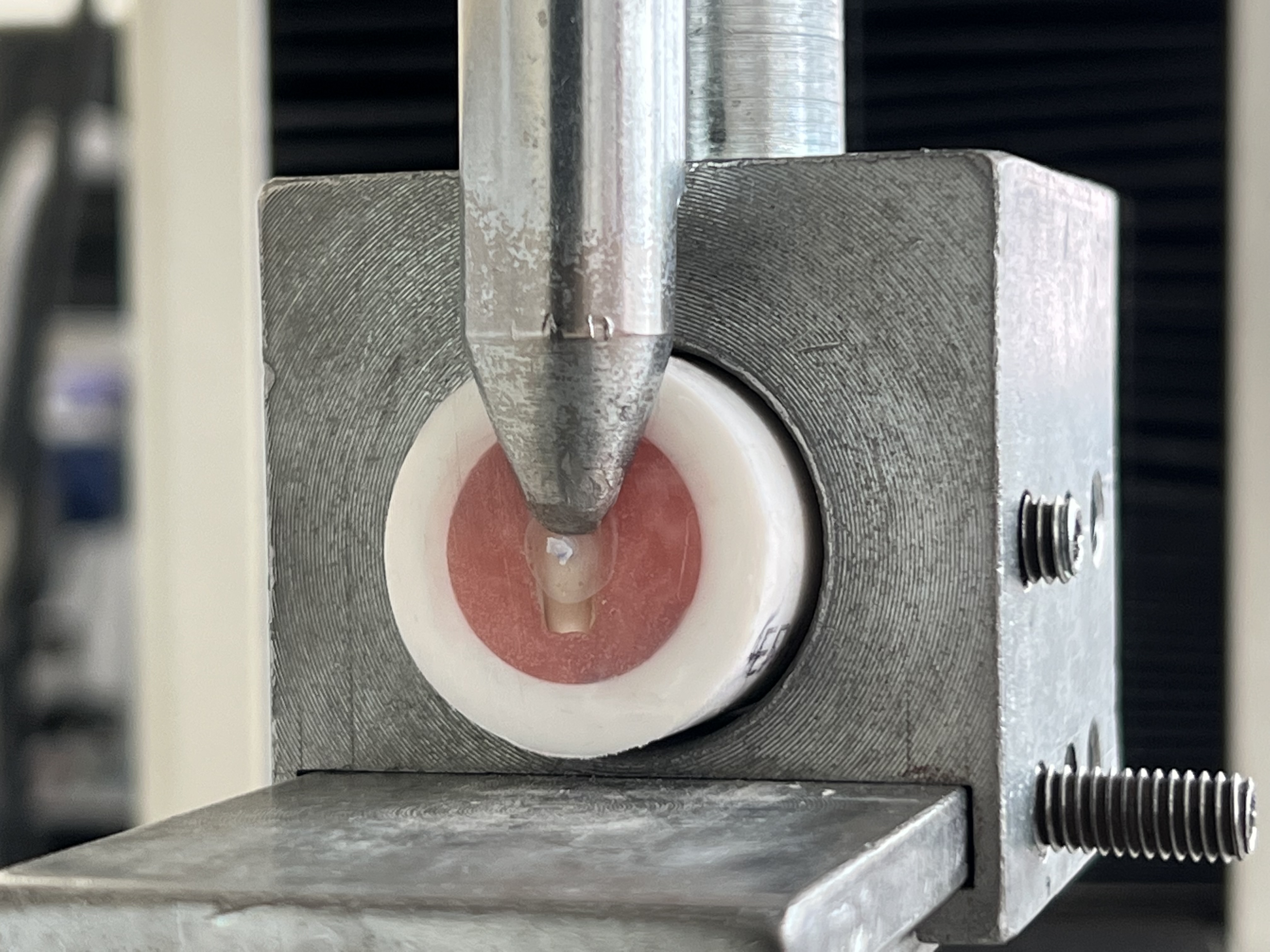

Supplement: Supplementary file 2 — Supplementary Material 2. [file 12903_2026_8010_MOESM2_ESM.jpeg]

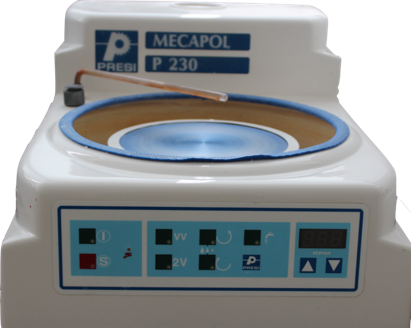

Supplement: Supplementary file 3 — Supplementary Material 3. [file 12903_2026_8010_MOESM3_ESM.png]

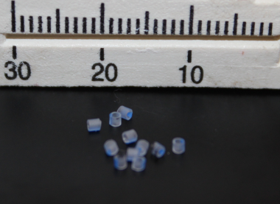

Supplement: Supplementary file 4 — Supplementary Material 4. [file 12903_2026_8010_MOESM4_ESM.png]

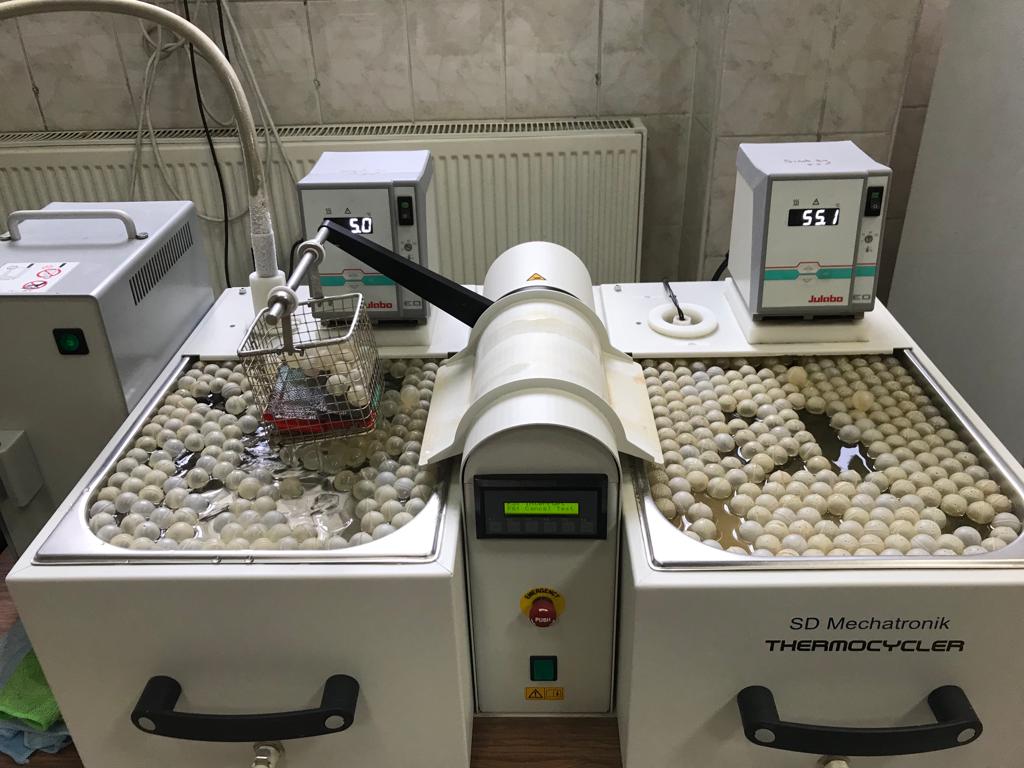

Supplement: Supplementary file 5 — Supplementary Material 5. [file 12903_2026_8010_MOESM5_ESM.jpeg]

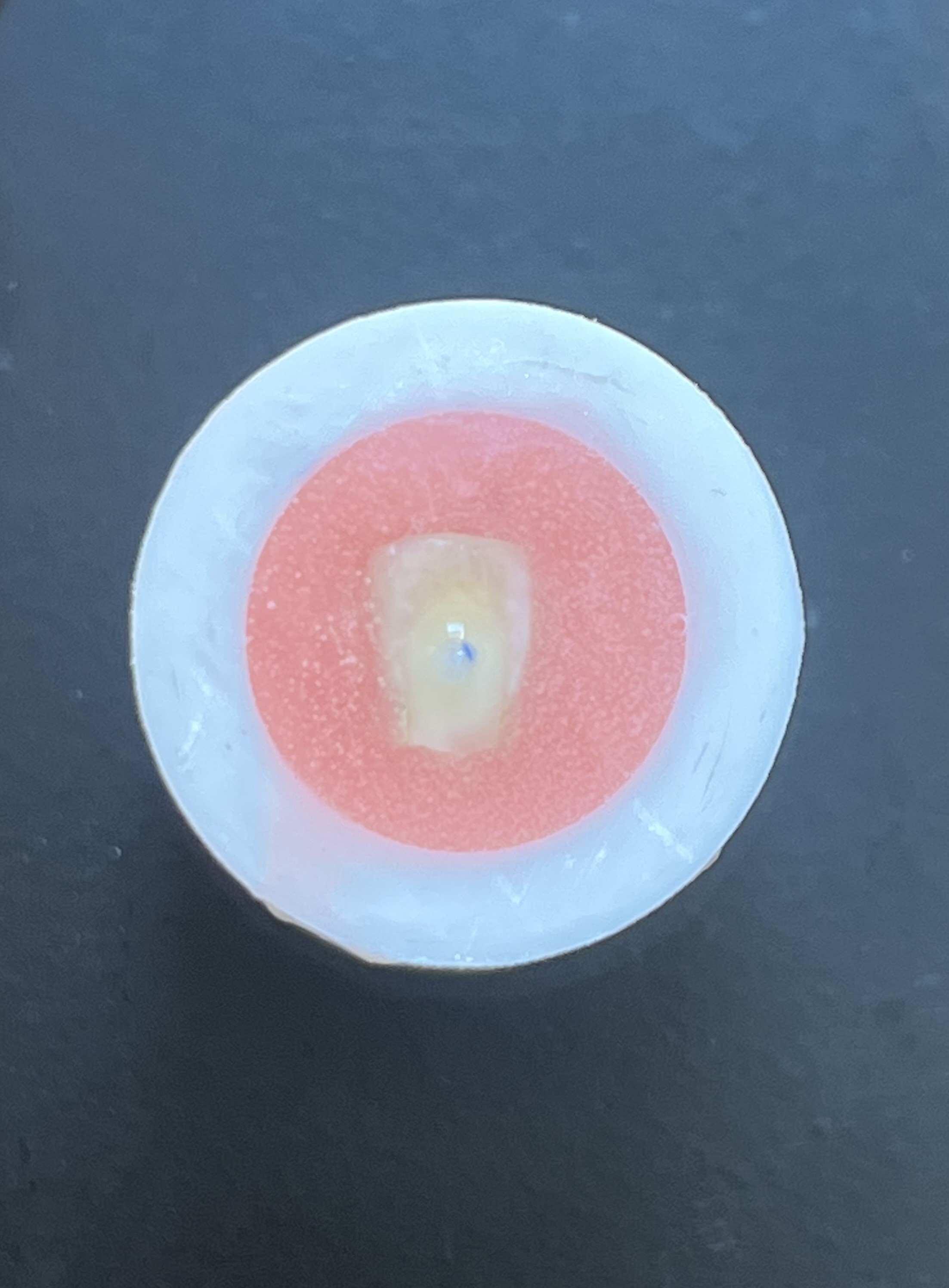

Supplement: Supplementary file 6 — Supplementary Material 6. [file 12903_2026_8010_MOESM6_ESM.jpeg]

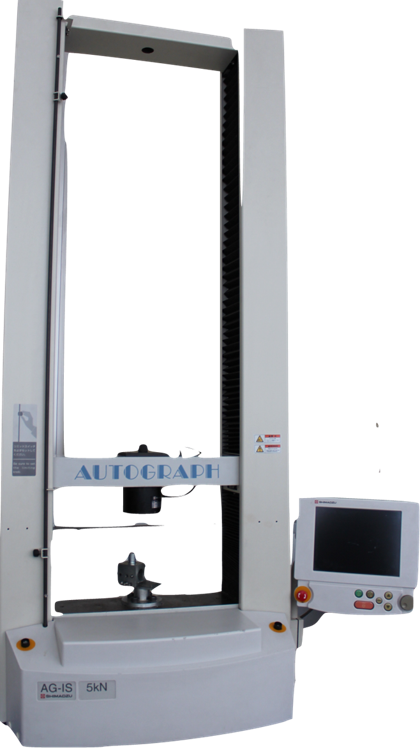

Supplement: Supplementary file 7 — Supplementary Material 7. [file 12903_2026_8010_MOESM7_ESM.png]
